# Adverse Effects of Proton Pump Inhibitors—Evidence and Plausibility

**DOI:** 10.3390/ijms20205203

**Published:** 2019-10-21

**Authors:** Reidar Fossmark, Tom C. Martinsen, Helge L. Waldum

**Affiliations:** 1 Department of Clinical and Molecular Medicine, Faculty of Medicine and Health Sciences, Norwegian University of Science and Technology (NTNU), Prinsesse Kristinas gate 3, 7006 Trondheim, Norway; 2 Department of Gastroenterology and Hepatology, St Olav’s Hospital—Trondheim University Hospital, Prinsesse Kristinas gate 1, 7030 Trondheim, Norway

**Keywords:** proton pump inhibitors, adverse effects, gastrin, gastric cancer, renal disease, liver disease, fracture risk

## Abstract

Proton pump inhibitors (PPIs) have been increasingly used over the last decades and there are concerns about overuse and the numerous reported side-effects. It is uncertain whether associations between PPI use and potential side effects are causal. However, important evidence from experimental and mechanistic studies that could support a causal relationship may have been underestimated by epidemiologists and meta-analysists. In the current manuscript we review the combined epidemiological and mechanistic evidence of the adverse effects of PPI use.

## 1. Introduction

Proton pump inhibitors (PPIs) are irreversible inhibitors of the gastric H^+^K^+^ATPase in parietal cells and they reduce acid secretion. PPIs have a short plasma half-life but bind irreversibly to proton pumps and new proton pumps must be synthesized before acid secretion is restored. The degree and duration of gastric hypoacidity caused by repeat PPI dosage exceed the effects of competitive histamine 2 receptor antagonists (H2RA) by far [1,2,3] and a phylogenetically well-preserved biological function [4] is nearly removed. PPIs have long been used in the management of acid-related gastrointestinal diseases, such as peptic ulcers and gastro-esophageal reflux disease (GERD). However, an increase in PPI use has been well described in many countries over the last decades [5,6]. This increase is perceived to be caused by the widespread use of PPIs in treatment of dyspepsia and prevention of gastrointestinal bleeding in patients prescribed antiplatelet therapy or non-steroid anti-inflammatory drugs (NSAIDs), coupled with the belief that PPIs have few adverse effects. Furthermore, even though the indications for PPI use have expanded, numerous studies have documented prevalent inappropriate PPI prescription [7,8,9], so that patients without indication may in many Western populations be the largest group of users. Side-effects of PPIs seem to be mainly caused by the desired drug effect, that is to induce gastric hypoacidity, which in turn may directly or indirectly cause harm. Short-term effects of PPIs are relatively well studied, but the consequences of long-term profound acid inhibition are not fully known, as the observation time in epidemiological studies is often too short for the detection of diseases that develop over many years. Another problem with epidemiologic studies is the inherent potential of unmeasured confounding factors. Therefore, we have emphasized essential translational research and mechanistic studies that may underpin observations in epidemiological studies. More importantly, mechanistic studies of long-term PPI use may be more significant than the relatively short, but large, epidemiological studies of patients using PPIs. This is particularly important when studying diseases with long latency, such as cancers. 

## 2. Results and Discussion

**Figure 1:**
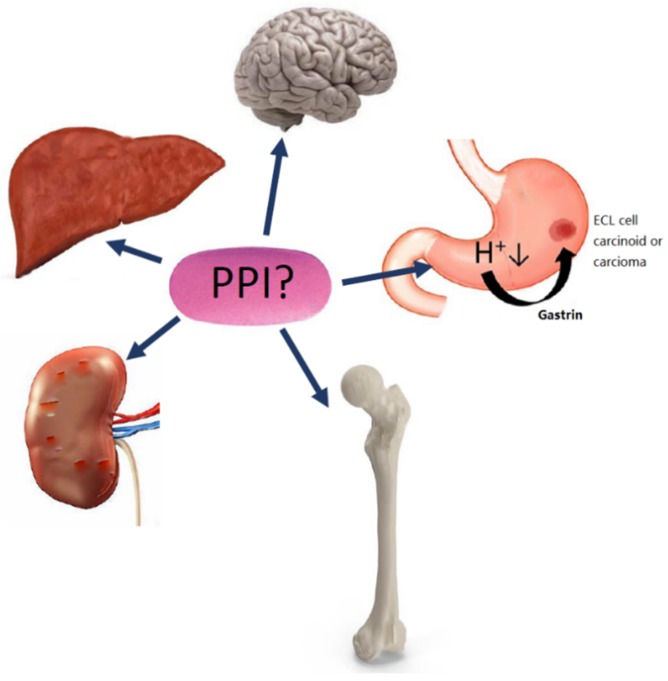
Numerous side effects of proton pump inhibitors (PPIs) have been proposed, including increased risk of gastric neoplasia, kidney disease, dementia, liver disease, and fractures.

Numerous types of side effects of PPIs have been proposed and we have in this review focused on the increased risk of gastric neoplasia, kidney disease, bone fractures, impaired absorption of micronutrients, dementia, and liver disease (Figure 1). The increased risk of infections is reviewed in a separate publication in this Special issue of IJMS (Martinsen TC et al., pending submission). Many of the side-effects reviewed are relatively rare in contrast to the widespread worldwide use of PPIs. Some of this may be explained by inter-individual variation in acid inhibition in humans and that a proportion of PPI users have more profound and long-lasting acid inhibition following ingestion, which is reflected by variations in serum gastrin concentrations [10,11]. Genetic variation in *CYP2C19* contributes to differences in the inhibitory activity of the PPIs. About 15–20% of Asians and 2–6% of Caucasians are known to be slow or poor metabolizers [12]. Patients with a *CYP2C19* genotype resulting in a poor metabolizer phenotype have a considerable higher median 24 h intragastric pH than extensive metabolizers [13]. Although direct evidence is absent it seems likely that poor metabolizers, who have more pronounced hypoacidity and hypergastrinemia, are more likely to develop many of the adverse effects discussed in this manuscript. Animal studies of the effects of PPIs and H2RA have often used doses per kg body weight that are much higher than the doses used in patients. This was done in order to examine the intended and therapeutic effect of PPIs in patients, which is induction of gastric hypoacidity, and rodents require much higher doses to achieve such effects. Consequently, many appropriately-designed animal studies have used high doses of PPIs, which are particularly important if negative findings are to be reported. Whereas rats become hypoacidic at doses of 400 µmol/Kg/day omeprazole, it is nearly impossible to administer a PPI to mice in a sufficient dose (1750 µmol/Kg/day subcutaneously) to induce 24 h profound hypoacidity and hypergastrinemia [14].

### 2.1. Gastric Neoplasia

The risk of gastric neoplasia in patients with gastric hypoacidity and hypergastrinemia was noted in patients with chronic atrophic gastritis decades before PPIs were invented [15]. Before PPIs were marketed for widespread use, it was known that rats given omeprazole (400 µmol/Kg/day) or the irreversible H2RA loxtidine (250–600 mg/Kg/day) in doses sufficient to inhibit gastric acid secretion and cause hypergastrinemia, developed enterochromaffin-like (ECL)-cell tumours in the gastric corpus [16,17]. Shortly thereafter it was realized that the competitive H2RA ranitidine also caused ECL cell tumours, when given the dose needed to achieve prolonged acid inhibition [18]. Further studies have demonstrated that ECL cell tumours in the corpus remnant may also be induced by partial corpectomy causing hypergastrinemia [19,20] or by administration of ciprofibrate, a drug that induces hypergastrinemia without altering gastric acidity [21,22,23]. Transgenic *INS-GAS* mice have hypergastrinemia accompanied by gastric hyperacidity and develop tumours in the gastric corpus with an adenocarcinoma phenotype [24]. Inoculation by *Helicobacter felis* increases the hypergastrinemia and accelerates the carcinogenesis considerably [24]. The rodent *Mastomys natalensis* develops tumours in the gastric corpus that were originally classified as adenocarcinomas [25,26]. However, the lesions were later re-classified as ECL cell tumours and the observed propensity may be caused by a mutation leading to constitutively activation of the CCK2/gastrin receptor [27]. The tumorigenesis in *M. natalensis* is enhanced by the H2RA loxtidine [28] and inhibited by the gastrin receptor antagonist YF476 (later named netazepide) [29], further demonstrating the role of gastrin. Male Japanese cotton rats given loxtidine in order to induce gastric hypoacidity develop tumours with an adenocarcinoma phenotype, but neuroendocrine differentiation, after six months [30]. The spontaneous tumour formation in Japanese female cotton rats may be prevented by the gastrin receptor antagonist YF476 [31]. Through the above-mentioned series of animal studies, it has been documented that long-term hypergastrinemia, whether accompanied by gastric hypoacidity or hyperacidity, causes neoplasia in the gastric corpus with varying expression of neuroendocrine markers, in all species where sufficient hypergastrinemia has been be achieved [32]. The pivotal role of the ECL cell in hypergastrinemia-driven carcinogenesis has been described in a separate paper in IJMS [33]. The trophic effect of gastrin in rats [34] as well as in patients with chronic atrophic gastritis and ECL cell dysplasia [35,36] seems to level off at values below 500 pM and studies suggest that the dose-response relationship of gastrin on its target cell is very similar in rodents and humans. The increased risk of gastric ECL neuroendocrine tumours (NETs) and carcinomas in PPI users has therefore been predicted since the 1980s [37]. The ECL cell is the target cell of gastrin and ECL cell carcinoids in PPI users, although probably underreported by some [38,39,40], and the tumours may regress after cessation of PPI use [41]. Furthermore, patients homozygous of an inactivating mutation in the H^+^K^+^ATPase alpha subunit develop ECL cell carcinoids and adenocarcinoma in their third to fourth decade [42,43]. These patients represent the human genetic disease that best models the consequences of long-term PPI use and also indicate that the duration of profound acid inhibition needed to cause gastric neoplasia, either ECL cell carcinoids or adenocarcinomas, in most patients could be several decades. In this context, it should be noted that carcinogenesis driven by hypergastrinemia in rodent models takes months to years before neoplasia is found, a timespan that could translate into decades in humans and this observation could be relevant when starting long-term profound acid inhibition in young individuals. Another human disease that causes gastric hypoacidity and hypergastrinemia similar to a proportion of patients using long-term high-dose PPIs, is autoimmune chronic atrophic gastritis. These patients have an increased risk of adenocarcinomas in the gastric corpus and fundus, which was noted in the 1950s [15] and the risks of both adenocarcinoma and ECL cell tumours were studied during the subsequent decades [35,36,44]. The annual risk of gastric adenocarcinoma seems similar to the risk of ECL cell tumour formation in such patients and is in the range of 0.3–0.5% [44,45]. More recently, it was reported that PPI users have an increased risk of gastric adenocarcinoma in epidemiological studies, with an overall standardized incidence ratio of 3.38 in a Swedish population [46], and in an additional publication it was emphasized that the risk did not decline with time [47]. In a Japanese population of patients who received *Helicobacter pylori* eradication, PPI users had an adjusted hazard ratio (HR) of 3.61 for gastric cancer during follow-up [48]. Furthermore, Cheung et al. studied a population in Hong Kong and found an increased risk of gastric cancer in patients given *H. pylori* eradication treatment with an HR of 2.44, and the HR increased with longer duration of PPI use [49]. H2RA users did not have higher risks of gastric cancer in any of these studies. Numerous studies suggest that hypergastrinemia, the common factor found in many conditions with increased cancer risk, is a key element in gastric carcinogenesis of the corpus and fundus [50,51]. PPIs cause gastric atrophy in a larger proportion of patients with GERD compared to those operated by fundoplication, particularly in *H. pylori*-positive individuals, where nearly one third developed atrophy during the five-year follow-up [52]. Prolonged acid inhibition also seems to preclude the recovery of atrophy following *H. pylori* eradication [53], which may contribute to persistent hypoacidity and hypergastrinemia even after eradication. Recently, self-reported adverse effects of PPI use were examined by using data from a randomized controlled trial (COMPASS) with over 17,000 participants whereof half the patients received pantoprazole [54]. The overall number of GI cancers was low during the three years of follow-up; the incidence of gastric cancer was not specified and incidence of atrophic gastritis was only 0.2% in three years [54]. This is in marked contrast to the incidence of atrophy in studies with gastric PPI-effects as a primary endpoint, where 19% of patients overall and 30.5% of *H. pylori*-positive subjects developed atrophic gastritis after five years [52], whereas a less targeted study design may preclude the evaluation of the effects of PPIs on gastric premalignant changes. It should be noted that the prevalence of premalignant gastric lesions at baseline may differ between patients in the follow-up studies of *H. pylori*-positive patients who for clinical reasons were tested for *H. pylori* and then received eradication treatment, and the COMPASS population who were given prophylactic PPIs. In a stomach with premalignant changes such as atrophy, metaplasia, and dysplasia, cancer may develop more rapidly than without such changes.

Other macroscopic changes in the gastric mucosa of PPIs use include fundic gland polyps [55,56], black spots, and cobblestone-like lesions [57] which are of uncertain clinical significance. However, a small proportion of patients develop fundic gland polyposis and this sub-group may require further evaluation. In total, numerous mechanistic studies suggest that hypoacidity and hypergastrinemia increase the risk of gastric cancer in the corpus/fundus and this was also supported by some epidemiological studies.

### 2.2. Renal Disease

It was first reported in 1992 that PPI use could cause acute interstitial nephritis (AIN) [58] and more than a decade later PPI-induced AIN became recognized as a clinical entity [59]. Due to their widespread use, PPIs are now considered to be among the most common causes of drug-induced AIN worldwide [60]. While one has estimated the mean time from starting PPIs to the clinical presentation of AIN to be 10 weeks, AIN may develop as late as nine months after treatment start and the risk is not dose dependent. More recently, several studies have reported an increased risk of incident chronic kidney disease (CKD) in PPI users (HR 1.18) [61], also after adjusting for uneven distribution of factors that could affect the risk of CKD (HR 1.76 compared with propensity score-matched non-users) [62]. The studies have some methodological weaknesses, including adjustments for baseline estimated glomerular filtration rate (eGFR) [63] and the general uncertainty related to residual confounding factors. In the COMPASS trial, baseline renal function was known, but patient-reported incident of CKD during follow-up did not differ between the study groups [54]. Incident renal failure that required treatment seemed likely to be self-reported during the study, whereas the incidence of asymptomatic modest reduction of renal function was more likely to be underreported [54].

The mechanism behind the observed increased risk of CKD in PPI users is obscure. It was initially considered whether drugs designed to inhibit the gastric proton pump also inhibit other proton pumps, such as the renal tubular proton pump (H^+^-ATPase) which in an in vitro tubule suspension from rats seemed to be affected by high concentrations of omeprazole [64]. However, as PPIs are weak bases with a pKa of 4, they are mainly activated in the acidic parietal cell canaliculi and bind covalently to the H^+^K^+^ATPase [65]. Furthermore, omeprazole does not affect electrolyte handling or acidification in urine in patients using 60 mg/day in the short term [66], and there is no evidence of PPI effects mediated by inhibition of the tubular proton pump in vivo in a clinical setting. Interestingly, it was recently reported that over half of patients who suffered PPI-induced AIN did not fully recover [67] suggesting that PPI-induced CKD may be due to progression of AIN, with inflammatory interstitial infiltrate and oedema, to chronic interstitial scarring and tubular atrophy. Altogether, there is good evidence that PPIs cause AIN and some evidence that they also increase the risk of CKD.

### 2.3. Fracture Risk

The risk of osteoporosis-related fractures in long-term PPI users became a concern after several observational studies reported a time- and dose-dependent increase in fracture risk [68,69,70]. In two recent meta-analyses of observational studies, PPI use was found to be significantly associated with an increased risk of hip fracture (RR 1.30), any-site facture (HR 1.29), and spine fracture (HR 1.49), which was not observed in patients with H2RA exposure [71,72]. Despite these studies, one has not been able to find a reduction in bone mineral density (BMD) during PPI treatment [73,74] and it was proposed that the observed increase in fracture risk is caused by increased comorbidity and lower BMD at the start of treatment. It was also found that PPI use was associated with a higher risk of recurrent falls in older women [75]. In a recent randomized placebo-controlled study, PPI users had increased markers of bone turnover (P1NP and CTX), but no reduction in BMD after 26 weeks of use [76]. In the mentioned COMPASS trial, PPI users did not have an increased risk of fractures [54]. It seems likely that the incidence of self-reported fractures is reliable, and this study strongly suggests that PPI use for three years does not increase fracture risk. 

However, findings from several animal models of gastric hypoacidity have supported a causal relationship between long-term PPI use and the previously observed increased fracture risk. Rats given long-term omeprazole 400 µmol/Kg/day for three months exhibited reduced bone mineralization [77]. CCKB (gastrin) receptor-deficient mice are hypoacidic with hypocalcaemia, secondary hyperparathyroidism, and reduced BMD and bone quality at one year of age [78]. Furthermore, the skeletal changes in CCKB receptor-deficient mice could be reversed by calcium supplementation [79], suggesting a mechanism somehow related to impaired calcium handling and uptake in the gastrointestinal tract. H^+^K^+^ATPase beta subunit-deficient mice are also hypoacidic, hypergastrinemic, display an elevated parathyroid hormone [80], and have reduced BMD and bone quality [80,81], which only slightly improved by administration of a gastrin receptor antagonist. Patients with chronic atrophic gastritis, a condition that may be analogous to long-term high-dose PPI use with respect to hypoacidity, hypergastrinemia, and absorption of micronutrients, have an increased risk of fractures [82,83], reduced BMD [84], and reduced calcium carbonate absorption in fasting state [85]. However, absorption of other calcium compounds and calcium carbonate ingested together with food seems unaffected by hypoacidity [85,86,87] and in a recent well-designed, randomized trial, PPI-use did not affect serum calcium, serum parathyroid hormone, or urinary calcium [76]. Although early in vitro studies of omeprazole suggested that it could decrease bone resorption [88], there has not been further convincing evidence that PPIs have direct in vivo effects on bone. A potential role of elevated circulating histamine has also been proposed based on knowledge about the gastrin—histamine sequence in regulation of acid secretion [89], the observation of osteoporosis in patients with hyperhistaminemia caused by systemic mastocytosis [90], the increased bone loss in histidine decarboxylase-deficient mice [91], and reduced fracture risk in PPI users also using a histamine 1 receptor antagonist (H1RA) [92]. However, a H1RA did not alter the osteoporotic phenotype in H^+^K^+^ATPase beta subunit-deficient mice [93].

In conclusion, although findings from several animal models and patients with chronic atrophic gastritis have suggested that gastric hypoacidity affects bone metabolism negatively, the recent data from a randomized trial [54] suggest that PPIs do not increase fracture risk. The lack of one plausible mechanism also weakens the hypothesis that PPI use cause increased fracture risk. The observed increased fracture risk in epidemiologic studies may be explained by residual confounding factors related to increased comorbidity and risk of falling.

### 2.4. Dementia

A few years ago, two epidemiological studies reported that PPI users had an increased risk of incident dementia during follow-up (HR 1.44) [94], whereas another study found an increased risk of all-cause dementia (HR 1.38) [95], and more specifically for Alzheimer’s disease (HR 1.44) [95]. Similar risks were also found in retrospective studies (OR 1.55) [96]. These positive epidemiological studies are supported by previous findings in animal studies where transgenic mice predisposed to accumulation of beta-amyloid administered lansoprazole 20–200 mg/Kg had dose-dependent accumulation of beta amyloid (Aβ40 and Aβ42) in the central nervous system [97]. Many researchers have also suggested prion-like mechanisms in Alzheimer’s disease [98,99], where beta amyloid and tau are the soluble components in formation of plaques and tangles, respectively. The role of gastric acid in the defence against transmissible degenerative encephalopathies has been examined in mice. Mice with gastric hypoacidity induced by administration of ranitidine in the drinking water were susceptible to infection by the scrapie agent 139A in a ranitidine concentration-dependent manner [100]. Furthermore, mice with short-term hypoacidity induced by omeprazole were also more likely to become infected after inoculation with a scrapie agent [101]. 

Altogether it seems plausible that the association between dementia and PPI use could be causal; the increased risk of dementia has not been reproduced in the following epidemiological studies [102,103]. The previously mentioned COMPASS trial did not find increased risk of dementia in PPI users [54], however, there are inherent limitations tied to recording patient-reported incident dementia and according to published calculations the study was not powered for detecting an OR of incident dementia in the same range as previous studies. In conclusion, while epidemiological studies are conflicting, preclinical studies support a possible causal relationship.

### 2.5. Liver Disease

PPI use has been claimed to increase the risk of the cirrhosis-related complications of hepatic encephalopathy (HE) and spontaneous bacterial peritonitis, as well as to influence the risk of cirrhosis and liver cancer. PPI use increases the risk of developing HE in patients with cirrhosis [104] as well as the severity of HE [105]. Two recent meta-analyses confirmed the association [106,107] and the proposed mechanism is that PPIs induce bacterial overgrowth in the intestine [108]. It is known that PPIs do not only change the bacterial composition in the stomach, but also in faeces with a significant shift towards oral flora in principal coordinate analyses (PCoA) [109,110], demonstrating that the entire gastrointestinal tract is affected downstream of gastric hypoacidity. PPI use has been identified as a risk factor for the development of spontaneous bacterial peritonitis (SBP), also in multivariate analyses adjusting for known risk factors including the severity of liver disease [111,112]. In H^+^K^+^ATPase alpha subunit-deficient mice as well as mice given omeprazole, alcohol-induced liver damage worsens, which was seen in relation to intestinal bacterial overgrowth and an altered intestinal microbial composition [113]. More specifically, hypoacidity caused expansion of *Enterococci*, which was hypothesized to worsen alcoholic liver disease in mice as well as in humans [113]. Furthermore, others have identified increased abundances of *Veilonella parvula* and *Streptococcus salivarius* in the faecal microbiome of cirrhotic PPI users to be associated with liver-related mortality during three years of follow-up [114]. 

The risk of liver cancer in PPI users was recently found to be increased in two different populations, whereas no such risk was seen in H2-blocker users [115]. The underlying mechanism may be similar to that of HE, SBP, and alcohol-induced liver damage, whereby bacterial overgrowth [116] results in increased portal venous concentrations of several proposed harmful substances, including secondary bile acids [117]. In general, there is considerable interest in the role of intestinal microbiome in the pathogenesis of chronic liver diseases [118] that cannot be reviewed in depth here. 

Although the epidemiological evidence is limited for the influence of PPIs on the pathogenesis of liver diseases including cancer, bacterial overgrowth and altered bacterial composition are indeed well-documented phenomena that raise concerns about adverse liver effects in PPI users. 

### 2.6. Micronutrient Deficiency

Numerous studies have documented that gastric acid influences the absorption of minerals ingested as salts and protein-bound vitamin B12.

#### 2.6.1. Vitamin B12

Increased risk of vitamin B12 deficiency has been a concern since absorption of protein-bound, but not unbound, vitamin B12 was found to be reduced during PPI use [119]. Observational studies suggested an increased risk with an OR of 1.65 for PPI users and OR of 1.25 for H2-blocker users [120]. However, a considerable number of other studies has not reproduced these findings and there seems to be insufficient evidence for routine measurements of B12 concentration in blood [121,122].

#### 2.6.2. Calcium

Profound acid inhibition may interfere with the absorption of calcium [123]. However, long-term PPI does not seem to reduce absorption of water-soluble calcium salts and calcium absorption from the diet [76,124] and this has also weakened the hypothesis of disturbed calcium metabolism as a mechanism causing increased fracture risk.

#### 2.6.3. Iron

Absorption of iron may be reduced in patients with gastric hypoacidity, as seen in patients using oral ferrous sulphate supplementation and omeprazole [125]. Interestingly patients with hereditary hemochromatosis have reduced absorption of dietary non-heme iron and PPI use also seems to reduce the need for phlebotomy [126,127]. Reduced absorption of iron was also observed in patients with hypoacidity due to chronic atrophic gastritis [128]. A large case-control study found PPI use to be associated with an increased risk of iron deficiency [129], but the magnitude of reduced iron absorption is most likely small in most individuals and the clinical importance has been questioned. 

#### 2.6.4. Magnesium

Hypomagnesemia has been described in PPI users since 2006 [130]. Many studies have suggested an increased risk and that hypomagnesemia develops in a proportion of PPI users [131]. Meta-analyses have been problematic due to study heterogeneity, but a recent analysis found that PPI users had an increased risk of hypomagnesemia (RR 1.44) compared to non-users [132], whereas analysis of only high-quality studies found an RR of 1.63 [133]. Hypomagnesemia is rare and it seems that mainly patients who already use a diuretic are at risk [131]. The mechanism of hypomagnesemia in PPI users is uncertain, but in depth reviews of case series suggest that reduced intestinal absorption rather than renal tubular loss is important [134]. In conclusion, symptomatic hypomagnesemia in PPI users is well-documented, but rare.

## 3. Conclusions

Epidemiological studies on PPI use and side effects may suffer from residual confounding and in some instances reverse causation. Furthermore, studies designed to detect long-term effects or relatively rare side effects have not been performed. Results from mechanistic studies, including animal studies, suggest that there are known and most likely unknown side effects of long-term gastric acid inhibition that should be considered when prescribing PPIs. Many patients have appropriate indications for long-term PPI use that may outweigh the risks discussed above. However, a large proportion of PPI users without indication have no benefits to outweigh any risk of side effects at all and this is a major concern. As some of the potential side effects may have an incubation time of years or even decades [42,43], the risks and benefits of starting long-long PPI use should be carefully considered. This general consideration regarding long-term medical treatment seems especially relevant in younger individuals who may initiate PPI use that could last 70 to 80 years. 

## Abbreviations

AIN	Acute interstitial nephritis
BMD	Bone mineral density
CCKB	Cholecystokinin B
CKD	Chronic kidney disease
CTX	carboxy-terminal collagen crosslinks
ECL	Enterochromaffin-like
eGFR	Estimated glomerular filtration rate
GERD	Gastroesophageal reflux disease
GI	Gastrointestinal
H1RA	Histamine 1 receptor antagonist
H2RA	Histamine 2 receptor antagonist
HE	Hepatic encephalopathy
*H. pylori*	*Helicobacter pylori*
HR	Hazard ratio
NET	Neuroendocrine tumour
NSAID	Non-steroid anti-inflammatory drug
OR	Odds ratio
P1NP	procollagen type 1 N-terminal propeptide
PCoA	Principal coordinate analysis
PPI	Proton pump inhibitor
RR	Relative risk
SBP	Spontaneous bacterial peritonitis
